# New Approaches Based on Non-Invasive Brain Stimulation and Mental Representation Techniques Targeting Pain in Parkinson’s Disease Patients: Two Study Protocols for Two Randomized Controlled Trials

**DOI:** 10.3390/brainsci11010065

**Published:** 2021-01-06

**Authors:** Yeray González-Zamorano, Josué Fernández-Carnero, Francisco José Sánchez-Cuesta, Aida Arroyo-Ferrer, Athanasios Vourvopoulos, Patricia Figueiredo, José Ignacio Serrano, Juan Pablo Romero

**Affiliations:** 1 Escuela Internacional de Doctorado, Department of Physical Therapy, Occupational Therapy, Rehabilitation and Physical Medicine, Universidad Rey Juan Carlos, 28933 Alcorcón, Spain; yeraygonzalezzamorano@gmail.com; 2 Facultad de Ciencias Experimentales, Universidad Francisco de Vitoria, 28223 Pozuelo de Alarcón, Spain; fjose.sanchez@ufv.es (F.J.S.-C.); aida.arroyo@ufv.es (A.A.-F.); 3 Department of Physical Therapy, Occupational Therapy, Rehabilitation and Physical Medicine, Universidad Rey Juan Carlos, 28933 Alcorcón, Spain; josue.fernandez@urjc.es; 4 Institute for Systems and Robotics-Lisboa, Department of Bioengineering, Instituto Superior Técnico, Universidade de Lisboa, 1049-001 Lisbon, Portugal; athanasios.vourvopoulos@tecnico.ulisboa.pt (A.V.); patricia.figueiredo@tecnico.ulisboa.pt (P.F.); 5 Neural and Cognitive Engineering Group, Centre for Automation and Robotics, Spanish National Research Council, 28500 Arganda del Rey, Spain; jignacio.serrano@csic.es; 6 Brain Damage Unit, Beata María Ana Hospital, 28007 Madrid, Spain

**Keywords:** Parkinson’s Disease, transcranial direct current stimulation, action observation, motor imagery, pain

## Abstract

Pain is an under-reported but prevalent symptom in Parkinson’s Disease (PD), impacting patients’ quality of life. Both pain and PD conditions cause cortical excitability reduction and non-invasive brain stimulation. Mental representation techniques are thought to be able to counteract it, also resulting effectively in chronic pain conditions. We aim to conduct two independent studies in order to evaluate the efficacy of transcranial direct current stimulation (tDCS) and mental representation protocol in the management of pain in PD patients during the ON state: (1) tDCS over the Primary Motor Cortex (M1); and (2) Action Observation (AO) and Motor Imagery (MI) training through a Brain-Computer Interface (BCI) using Virtual Reality (AO + MI-BCI). Both studies will include 32 subjects in a longitudinal prospective parallel randomized controlled trial design under different blinding conditions. The main outcomes will be score changes in King’s Parkinson’s Disease Pain Scale, Brief Pain Inventory, Temporal Summation, Conditioned Pain Modulation, and Pain Pressure Threshold. Assessment will be performed pre-intervention, post-intervention, and 15 days post-intervention, in both ON and OFF states.

## 1. Introduction

Parkinson’s Disease (PD) affects between 4.1 and 4.6 million people in the world [1]. The diagnosis of PD is currently clinical and based on its motor manifestations (bradykinesia, rest tremor and rigidity). However, non-motor symptoms such as pain, fatigue, and neuropsychiatric manifestations are present in more than 70% of subjects [2].

Pain affects about 85% of patients [3] but is paradoxically under-reported [4] and consequently under-treated in PD patients with a great impact in their quality of life [5]. Levodopa, which is the election treatment in PD, has shown controversial results regarding pain sensitivity [6,7] and has been shown to be ineffective for enhancing the endogenous pain modulation system [8]. Furthermore, there is a lack of management protocols and nonpharmacologic treatments for pain in PD [9].

Several syndromes are hypothesized to be involved in PD pain generation [10]. Generally, PD patients suffer from alterations in peripheral transmission, sensitive-discriminative processing, pain perception and pain interpretation in multiple levels, due to neurodegenerative changes in dopaminergic pathways, and non-dopaminergic pain-related structures [11]. Therefore, central mechanisms are proposed to be crucial for the development and establishment of pain in PD patients [12].

Regarding to pain processing features, PD patients have reduced pain thresholds [7], an augmented Temporal Summation (TS) after repetitive nociceptive stimulus [13] and the impairment of their Conditioned Pain Modulation (CPM) is correlated with a greater severity and premature onset of the disease [8].

Cortical excitability reduction is common in patients with pain [14]. Therefore, diverse therapies are being developed to counteract this cortical excitability reduction and consequently obtain effective pain relief [15,16]. In consonance with these findings, in PD condition, especially when levodopa is not acting (OFF state), there is also evidence of a decrease in cortical excitability [17]. However, PD patients have also shown to present an excessive corticospinal output at rest, associated with reduced intracortical inhibition, and impairments of intracortical facilitatory inputs during movement preparation and execution. Consequently, PD patients reveal motor cortex disfunction, which could be a valid target for therapeutic neuromodulation [18]. To the best of our knowledge, there are no studies targeting cortical excitability to treat pain in PD.

Thus, the present study protocol proposes two innovative non-pharmacologic approaches for the treatment of pain in PD patients, which incorporate current knowledge on physiological mechanisms and the clinical results of neuromodulation therapies used in other pain populations. Both protocols are based on neuromodulation, one of them through non-invasive brain stimulation and the other through mental representation techniques. Both protocols will be compared with respective placebo control groups, in order to evaluate their therapeutic effect, eliminating systematic errors such as expectations-related influences in the effect of the therapy as well as its potential adverse effects.

The non-invasive brain stimulation therapy will be transcranial direct current stimulation (tDCS) over the Primary Motor Cortex (M1). A tDCS over M1 is capable of increasing corticospinal excitability in both M1 and other pain processing-related areas such as thalamus, Dorsolateral Prefrontal Cortex (DLPFC), cingulate cortex and insula [15,16], also involved in PD pain processing [12]. These increments of cortical excitability have been correlated with pain relief in chronic pain such as fibromyalgia [19], osteoarthritis [20], migraine [21], and spinal cord injury [22]. It is also hypothesized that tDCS would be an effective strategy to treat central sensitivity-related pain [23], a process whose features are common with PD condition [3]. Moreover, specifically in PD, tDCS over M1 have shown to increase cortical excitability, augmenting the Motor Evoked Potential (MEP) amplitude by 78.5%, correlating with motor improvements [24]. The altered descending inhibitory systems in chronic pain could also be improved with high frequency repetitive transcranial magnetic stimulation, by increasing cortical excitability [25].

For the most part, the hypoalgesic effects generated by tDCS are due to the adjustment of its effect on the Primary Motor Cortex (M1). It is suggested that tDCS on M1 could reduce pain by increasing excitability, not only of the stimulated area, but also of other structures involved in pain processing, both sensory and emotional, such as the thalamus, dorsolateral prefrontal cortex (DLPFC), cingulate cortex, insula, and brain stem [15,16], thanks to cortical-subcortical connectivity [26].

Due to the coincidence between many of the most influential areas of pain in PD [12] and the areas excited by tDCS on M1, with these increases in excitability correlating with decreases in pain [15,16], there is a possible beneficial interaction between tDCS on M1 and pain in PD.

The mental representation techniques included in the protocol will be Action Observation (AO) and Motor Imagery (MI). In the same way, the combination of AO and MI has shown to synergically increase cortical excitability [27], influencing the activation of cortical areas such as M1 and DLPFC [28]. Specifically in PD, they also have demonstrated to produce corticomotor facilitation [29]. In addition, mental representation training can produce neurophysiological activity similar to actual exercise training [30], which have shown to decrease the intensity and severity of pain in PD patients [31].

There are no studies correlating effects of pain non-invasive neuromodulation with complete neuropsychological evaluation including computerized reaction time tasks and extensive neurophysiological measures such as direct cortical excitability evaluation using TMS and connectivity based in EEG entropy analysis. All these features have been described to be altered in PD and Pain.

The main aim of this study is to conduct two independent parallel randomized controlled trials based on both neuromodulation techniques targeting changes in 1. validated general and specific PD related pain scales 2. psychophysical measurements of pain modulation mechanisms and 3. wide neuropsychological and neurophysiological evaluation. Our main hypothesis is that both active neuromodulation techniques will be superior to their respective control placebo interventions. If this hypothesis is confirmed a further analysis of superiority will be proposed among both protocols.

## 2. Materials and Methods

The SPIRIT 2013 checklist has been used to assure the quality of the protocol [32].

### 2.1. Study Design and Participants

The participants will be recruited in PD outpatient’s clinic of the Beata María Ana Hospital of Madrid, Spain. The subjects included will be assessed by a neurologist expert in movement disorders. All patients will be idiopathic PD patients diagnosed according to the UK Parkinson’s Disease Society Brain Bank criteria who suffer from whatever PD-related pain, determined by The Parkinson’s Disease Pain Classification System (PDPCS) [33] and with a pain intensity of 3 out 10 points in the Visual Analogue Scale (VAS) while in ON state, that is, when levodopa is acting. The rest of specific inclusion criteria and the exclusion ones are listed in Table 1 and Table 2. During the protocol, changes in analgesic medication during in the previous week or PD treatment in the previous month will be forbidden and if necessary, will lead to exclusion of the patient. Informed consent for all included procedures (therapies and outcomes measurement), approved by the ethics committee, will be obtained from all subjects before their inclusion, and both studies will follow the principles outlined in the Declaration of Helsinki.

Sensitive data for all participants will be registered separately, anonymized, and guarded according to current data protection European laws. All data will be registered and double checked in a database designed specifically for the studies.

Recruitment in both protocols will be made so placebo and active groups will be matched by age and the recruitment will be stratified in groups of subjects younger than 60 years and older than 60, always ensuring that there is an equitable number of subjects in the two sections.

It is well known that there are gender differences in pain thresholds in healthy humans [34] and some evidence suggests that greater disease severity, female gender and higher doses of mediation are associated with greater pain intensity in PD [35]. For this reason, inclusion of at least one third of female subjects will be assured. Disease severity and total dopaminergic daily dose will be controlled in the final analysis of results.

#### 2.1.1. tDCS-Based Study

This study will be a triple-blinded experimental longitudinal prospective randomized controlled trial with a parallel design. The randomization will be realized through the GraphPad software (GraphPad Software, San Diego, C.A., U.S.A.), by an independent investigator. All the participants who fulfill the inclusion criteria and have none of the exclusion one will be randomly allocated in two groups: active-tDCS (a-tDCS) or sham-tDCS (s-tDCS). Allocation concealment will be ensured by the inclusion of the assigned group in closed opaque envelopes that will be opened at the time of the intervention. Triple-blind criteria will be achieved by identic collocation of the electrodes in both groups and by activating the “double-blind” option in the Starstim tCS^®^ Software (Neuroelectrics Inc, Barcelona, Spain) that allows the protocol to be concealed by writing a neutral number. The investigator in charge of concealing allocation will also conceal the protocols with the neutral number and the therapist will read it in the envelope, ignoring which number coincides with each intervention. At the end of the full treatment, patients will be asked about which stimulation condition they think they received and how confident they are in their guess. After each session a tDCS adverse effects questionnaire will be applied, the patient will be asked about any symptoms that they have experienced during the session in order to be able to identify possible adverse effects not described. In addition, their comfort will be assessed using a specific comfort questionnaire specified in the protocol section. These questionnaires will be necessary to obtain quantitative information on tDCS tolerability and blinding maintenance, which is scarce in PD patients [36]. Patients recruited will not meet in waiting rooms to avoid them to comment on their experience during the protocol. The statistician will be also blinded through the mentioned neutral numbers. Unblinding will be permissible in any event where it could pose a risk for the patient’s health. The Institutional Research Committee and Institutional Ethics Committee of Hospital Universitario 12 de Octubre approved this study with code 20-515 on December 2020 and the trial was registered in www.clinicaltrials.gov number NCT04651699.

#### 2.1.2. AO+MI-Based Study

This study will be a double-blinded experimental longitudinal prospective randomized controlled trial with a parallel design. The randomization will be realized through the GraphPad software (GraphPad Software, Inc CA 92037 EE.UU.). All the participants who fulfil the inclusion criteria and have none of the exclusion one will be randomly allocated in two groups: AO+MI through a Brain-Computer Interface (BCI) training paradigm in Virtual Reality (VR) (or neurogame) (AO+MI-BCI) or AO of non-related with movement illustrations (AO-control). Allocation concealment will be ensured by the inclusion of the assigned group in closed opaque envelopes that will be opened at the time of the intervention. Double-blind criteria will be achieved by following the same protocol with the same instruments in both groups. The evaluator will not be able to stay in the same room during the intervention and patients will not know the instructions and specific hypothesis of each protocol. The statistician will be blinded through the assignment of neutral numbers to both groups. Patients recruited will not meet in waiting rooms to avoid them to comment on their experience during the protocol. Unblinding will be permissible when any event could suppose a risk for the patient’s health. The Institutional Research Committee and Institutional Ethics Committee of Hospital Universitario 12 de Octubre approved this study with code 20-514 on December 2020 and the trial was registered in Clinical Trial, NCT04651478.

### 2.2. Intervention Protocols

In both studies, PD patients, independently of the group allocation, will undergo 10 sessions in two weeks (Monday to Friday). All sessions will be performed during ON state of patients and with their usual analgesic medication, to ensure their comfort.

#### 2.2.1. tDCS-Based Study

The Starstim tDCS^®^ stimulator (Neuroelectrics, Barcelona, Spain) will be used by an experienced physical therapist to transfer the direct current by a saline-soak pair of surface sponge electrodes (35 cm^2^).

In the experimental group (a-tDCS), the anode electrode will be placed over C3 (EEG 10/20 system) and the cathode electrode over the contralateral supraorbital area (Fp2), in order to enhance excitability of M1 [37]. Regarding the stimulated hemisphere, contralateral M1 will be stimulated in patients with asymmetric pain [38] but in patients with symmetric pain, the left hemisphere will be targeted due to the widespread changes induced by tDCS in other cortical areas, including contralateral M1 [26]. A constant current of 2 mA intensity (subthreshold intensity) will be applied for 20 min [39], with 30 s of ramp-up and 30 s of ramp-down.

For the sham stimulation (s-tCDS), the electrodes will be placed in the same positions as for M1 stimulation, but only applying ramping active current for 30 s in the beginning and at the end of the procedure for a reliable blinding [40].

During interventions, all subjects will be seated in a relaxed position in spite of this technique have demonstrated to be safe and pleasant [41]. When “on demand” analgesic medication is part of the usual treatment of the patient, the daily doses used will be registered to follow the short-term efficacy of the protocol. Our protocol follows the security and replicability recommendations for the use of this technique [42]. Possible adverse effects [41] will be assessed after each session by the Comfort Rating Questionnaire (CRQ), a modified version of the unpublished questionnaire from the Göttingen study group [43,44], and its apparition could lead in the interruption of the intervention. Although no severe adverse effects are expected. Any adverse effect will be notified to a licensed medical doctor. Management of possible adverse effects will be individualized and according to the severity. In case of headache or scalp pain, an extra dose of analgesics will be prescribed if necessary and recorded for each patient, in case of skin local reactions such as reddening or itching local application of talcum will be suggested. In case of sleep changes, concentration problems, mood changes, or any other non-previously described adverse effects detected in the daily post treatment assessment will be managed individually and in case of persistence the medical doctor will make the decision to suspend the protocol for the patient and set a follow-up until the symptoms are resolved.

#### 2.2.2. AO+MI-Based Study

The experimental group will follow three stages to complete each session:

Firstly, an experienced technician will acquire EEG through a BCIs system with 32 active electrodes equipped with a low-noise biosignal amplifier and a 24-bit A/D converter at 256 Hz (LiveAmp 8 biosignal amplifier, Brain Products GmbH, Gilching, Germany). The spatial distribution of the electrodes will cover primarily the motor and somatosensory areas of the brain. Specifically, the Frontal-Central (FC3, FC4), Central (C1, C2, C3, C4), and Central-Parietal (CP3, CP4) in a small Laplacian configuration for spatial filtering. EEG data acquisition and processing will be performed through the OpenVibe platform [45], which will transmit the data to the RehabNet Control Panel (Reh@Panel) [46] via the Lab Streaming Layer (LSL) (https://github.com/sccn/labstreaminglayer) protocol to control the virtual environment.

Secondly, the BCIs training protocol will be designed and adapted based on the Graz-BCI paradigm [47]. The first step will be the acquisition of the raw EEG data in order to extract features to train a classifier to distinguish Right and Left imagined hand movements. Thus, the patient will have to perform mental imagery of the corresponding hand, based on the presented stimuli in the screen. The training session will be configured to acquire data in 24 blocks per class (Right- or Left-hand imagery) in a randomized order. Afterwards, the data will be filtered both spatially and temporarily between the Alpha and Beta bands (8–30 Hz) for creating the feature vector to be used to train a Linear Discriminant Analysis (LDA) classifier.

Finally, patients will undergo the treatment itself through the NeuRow platform [48]. NeuRow is a gamified BCI training paradigm in VR (or neurogame) that allows patients to perform the same actions as they would do in real-life. In NeuRow, patients will see a boat and two high fidelity virtual arms gripping two oars in first person view. Patients will have to imagine the movement of each corresponding hand to rotate each oar and progress, observing the movement imagined on screen. The goal of the task is to collect as many flags as possible in a fixed amount of time. In order to improve adherence, the number of flags collected will be recorded in each session. It will be able to adapt the boat speed, turn speed and cut-off angle, to help patients not to deviate in excess from the target. The treatment itself will be performed for 20 min each session [49], divided in 4 series of 5 min to prevent fatigue. It is considered a multiplatform virtual environment developed in Unity game engine (Unity Technologies, San Francisco, CA, USA) and it has been used already with patients suffering from chronic motor disability [50].

The control group will follow an AO through non-related with movement illustrations protocol each session. The initial protocol will be omitted for being unnecessary. The same configuration will be applied simulating a BCI task, but playing a video about graphic illustrations, people faces and landscapes, never related with human movement. They will address interesting and changing topics to avoid patient’s boredom [51]. The control session will last 20 min, also divided in 4 series of 5 min and the therapist will give the instructions of observe and relax.

During interventions, all subjects will be seated in a relaxed position and whichever severe adverse event could lead in the interruption of the intervention, although they are considered unlikely. When “on demand” analgesic medication is part of the usual treatment of the patient, the daily doses used will be registered to follow the short-term efficacy of the protocol.

Possible adverse effects such as dizziness, mental fatigue, or whichever will be assessed and specifically managed after each session. Any adverse effect will be notified to a licensed medical doctor, although severe adverse effects are not expected, if they appear, the medical doctor will make the decision to suspend the protocol for the patient and set a follow-up until the symptoms are resolved.

### 2.3. Outcomes Measurement

**Figure 1:**
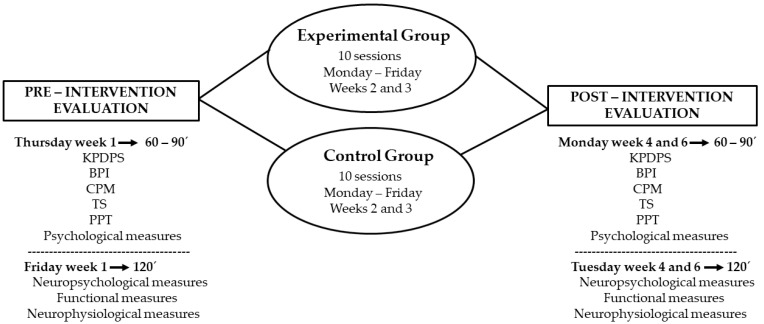
Schematic representation of both tDCS-based study and AO+MI-based study experimental procedures. It includes the days of the week and duration of each block of outcomes measurement in pre- and post-evaluation (KPDPS, King’s Parkinson’s Disease Pain Scale; BPI, Brief Pain Inventory; CPM, Conditioned Pain Modulation; TS, Temporal Summation; PPT, Pain Pressure Threshold; Psychological measures: Thursday/60–90 min; and Neuropsychological, Functional, Neurophysiological measures: Friday/120 min) and each treatment option (Experimental or Control group: Monday to Friday/10 sessions with the duration of each specific protocol, described in intervention protocols).

The same outcomes will be included in both studies. All of them will be assessed by a trained physical therapist in all groups the previous week, the following week, and 15 days after the 10 intervention sessions. The procedure is presented in Figure 1.

Every outcome will be measured in the ON state of PD patients, 1 h after taking their dopaminergic medication, to ensure their comfort. Nevertheless, Kings Parkinson’s Disease Pain Scale (KPDPS), CPM and TS will be also measured in OFF state, 30 min before medication intake, to assess the effects of both neuromodulation therapies when dopaminergic medication is not acting.

#### 2.3.1. Main Outcomes

##### King’s Parkinson’s Disease Pain Scale (KPDPS)

The KPDPS is a PD specific scale that evaluates the localization, frequency, and intensity of pain. It contains 14 items distributed in 7 domains: 1. Musculoskeletal Pain; 2. Chronic Pain; 3. Fluctuation-related Pain; 4. Nocturnal Pain; 5. Oro-facial Pain; 6. Discoloration, Oedema/Swelling Pain; 7. Radicular Pain. Each item is scored by severity (0, none to 3, very severe) multiplied by frequency (0, never to 4, all the time) resulting in a subscore of 0 to 12, the sum of which gives the total score with a theoretical range from 0 to 168. The KPDPS is considered a valid and reliable scale, with a Chronbach’s Alpha = 0.99 [52]. In addition, it is the unique validated scale in the assessment of pain in PD patients [53].

##### Brief Pain Inventory (BPI)

The BPI-short form will be used. It contains 15 items, including 2 multi-item scales to measure the intensity of pain and its impact on the function and welfare of patients. It also presents open questions to assess the localization of pain and the treatment used for its management, just as its effectiveness. The BPI is a valid and reliable scale, considered precise for the evaluation of severity of pain, easy to administer and interpret and applicable to diverse pain etiologies [54]. Its short form has been considered “recommended with caution” for the assessment of pain in PD patients, fulfilling every required criteria [53].

##### Pain Pressure Threshold (PPT)

PPT will be assessed with a handheld pressure algometer (FPX Model, Wagner Instruments, Greenwich, CT, USA). Two PPTs will be measured, one over the most painful area (peripheric hyperalgesia) and the other one over the middle of the distal phalanx of the thumb (central hyperalgesia). The PPT will be applied with the algometer perpendicular to the skin increasing at a rate of 1 kg/s [34] until the first sensation of pain. 3 measures with 30-s rest between them will be performed, taking the average as PPT. The algometry reliability to assess the PPT have demonstrated to be good-excellent, with an intraclass correlation coefficient of 0.84–0.96 [55].

##### Temporal Summation (TS)

The TS phenomenon, which represents excitatory modulation processes, will be generated through the application of 10 pulses of the handheld pressure algometer over the middle of the distal phalanx of the thumb with the intensity of the PPT, previously calculated [56]. In each pulse, pressure intensity will be increasing at a rate of 2 kg/s over the previously determined PPT intensity, leaving an interstimulus interval of one second according to the optimal method reported for inducing TS with pressure pain [57]. Before the first pressure pulse, subjects were taught to use a verbal numeric pain rating scale (VNPRS) to rate the pain intensity of the 1st, 5th, and 10th pressure pulses. The VNPRS ranges from 0 (“no pain”) to 10 (“the worst possible pain”) [58].

##### Conditioned Pain Modulation (CPM)

The CPM task, which assess the descending pain modulatory system, will be separated 5 min from TS task to prevent contamination. The PPT will be assessed in the middle of the distal phalanx of the thumb with the previously mentioned handheld algometer, corresponding to the first test stimulus. Afterwards, the patient will immerse the contrary hand up to the wrist into stirred ice-cold water (0–4 °C) maintaining it for 3 min, corresponding to the conditioning stimulus. If the pain is unbearable before the 3 min, the patient will be able to remove his/her hand. Immediately after removing the hand, a second PPT measure will be performed in the same place as the first one, corresponding to the second test stimulus [59]. After 1-min rest, a third PPT will be measured to assess the CPM residual functioning. The combination of PPT induced by handheld pressure algometer as test stimulus and cold water as conditioning stimulus are considered to be the most reliable method to assess CPM [60].

#### 2.3.2. Secondary Outcomes

##### Psychological and Functional Outcomes

The emotional outcomes assessed will be depressive symptoms, measured by Beck Depression Inventory (BDI), anxiety, measured by Stait-Trait Anxiety Inventory (STAI) and fear of movement-related pain, measured by Tampa Scale of Kinesiophobia (TSK-11). Cognitive outcome assessed will be catastrophizing thinking, measured by Pain Catastrophizing Scale. Finally, the functional outcome assessed will be the Unified Parkinson’s Disease Rating Scale (UPDRS), the most used scale for the assessment of disability in PD patients.

##### Neuropsychological Outcome

Reaction Times (RT) task will be performed through 2 related subtasks using a 15-inch monitor, controlled by Presentation^®^ software (http://www.neurobs.com).

The first subtask will be the Finger Taping (FT) task, to measure motor function [61]. This task is very sensitive to the slowing down of responses [62] and it has been also used as a measure of motor speed in PD patients [63]. In this task, following the Strauss application norms [62], the participants will be instructed to press the spacebar on the keyboard as fast as possible and repeatedly with the index finger. Five 10 s attempts will be performed with the dominant hand. The average time between two consecutive taps in the five trials will be the dependent variable.

Following this, the Simple Reaction Time (SRT) task will be performed to measure simple perception and sustained alertness [64]. Participants will be instructed to press the left mouse button as fast as possible when the stimulus “+” appears in the center of the screen at a size of 2 cm × 2 cm. The order of appearance will be constant for all participants. The task will consist of 50 trials lasting 2–3 min [65].

##### Neurophysiological Outcomes

The excitability of pain-related pathways will be measured by Transcranial Magnetic Stimulation (TMS) markers. Firstly, single pulse TMS will be performed to acquire resting motor threshold (rMT), motor evoked potentials (MEPs) and cortical silent period (CSP). Magstim Rapid2 device with a figure-of-eight magnetic stimulator coil will be used. For all assessments, the magnetic stimulator coil will be placed firstly on the right and secondly on the left M1 with a 5 min rest interval, and surface electromyogram from the contralateral first dorsal interosseous muscle will be recorded. TMS used amplitudes and EMG recordings will be recorded and stored in a computer for off-line analysis [66]. Stimulation output used to reach the rMT, MEP amplitudes and silent periods duration will be taken as outcomes.

Brain activity will be measured by Electroencephalography (EEG). An actiCHamp amplifier (Brain Vision LLC, Morrisville, NC, USA) will be used to amplify and digitize the EEG data at a sampling frequency of 512 Hz. The EEG data will be stored in a PC running Windows 7 (Microsoft Corporation, Washington, DC, USA). EEG activity will be recorded from 64 positions with active Ag/AgCl scalp electrodes (actiCAP electrodes, Brain Vision LLC, Morrisville, NC, USA). The ground and reference electrodes will be placed on AFz and on FCz, respectively Electroencephalography acquisition will be carried out by NeuroRT Studio software (Mensia Technologies SA, Paris, France). The EEG signal processing procedure will be performed using MATLAB functions (MathWorks Inc., Natick, MA, USA), specifically the EEGLab toolbox. The EEG registers performed will be resting (1 min), while FT task (30 s each hand, beginning with the dominant one), while wrist-extension task (5 min each hand, beginning with the dominant one) and with eyes closed (5 min). The participants will be asked to relax in the resting condition; the investigator will ensure they do not fall asleep, especially in resting and in eyes closed registers. Spectral entropy correlation, coherence and interhemispheric divergence differences among experimental group and controls will be analyzed as outcomes.

### 2.4. Sample Size Calculation

#### 2.4.1. tDCS-Based Study

The simple size for the tDCS-based study was calculated using G*Power software (version 2.1, Heinrich-Heine-Universität Düsseldorf, Düsseldorf, Germany), taking into consideration the design of two intervention groups and three assessment times. The primary outcome was chosen to be King’s Parkinson’s Disease Pain Scale with an estimated effect size of F = 0.25. An α level of 0.05 and a power of 90% was assumed, with a total sample of 28 subjects estimated. Considering a 15% follow-up loss, we estimated a minimal sample size of 32 patients, with a minimal recruitment of 16 required for each of the intervention groups.

#### 2.4.2. AO+MI-Based Study

The simple size for the AO+MI-based study was calculating taking into consideration the same parameters as tDCS-based study. It being estimated the same sample size of 32 patients.

### 2.5. Data Analyses

The SPSS software package (version 25.00; SPSS inc., Chicago, IL, USA) will be used by a blinded statistician for statistical analysis of the data. Demographic and clinical variables will be presented as mean and standard deviation. A 95% confidence interval (CI) will be taken, considering statistically significant all those values that had a *p* < 0.05. Parametric statistical tests will be used for hypothesis testing (*n* > 30). To show differences in the main variables, an analysis of variance (ANOVA) 2 × 3 of repeated measurements will be carried out taking time and group as factors, being the groups active-tDCS (a-tDCS) vs. sham-tDCS (s-tDCS) in the tDCS-based study and AO+MI-BCI vs. AO-control AO+MI-based study, and the measurements times pre-treatment, post-treatment, and at 15 days post-treatment. If significant differences are found in any of the interactions, a post hoc analysis will be performed with Bonferroni correction. The effect sizes will be calculated by means of the d of Cohen, being classified as small (d between 0.20 and 0.49), medium (d between 0.50 and 0.79), or large (d greater than 0.80) following the method of Cohen.

For the rest of the psychological variables, a Pearson’s correlation with the physical variables will be carried out in order to find relationships between both. Low correlation will be taken as that with values between 0.2 and 0.39; the correlation will be considered moderate when the value goes from 0.40 to 0.79; and high when the value of the correlation is greater than 0.80.

For the analysis of neurophysiological variables rMT, MEP magnitudes will be determined by averaging peak-to peak amplitudes. The ratio of the rMT, MEP amplitude recorded at each post-intervention time-point relative to the baseline rMT will be calculated [(MEP amplitude at each stage–baseline MEP amplitude)/baseline MEP amplitude Å~100]. Statistical analyses will be performed on the calculated test-to baseline MEP ratios. For the CSP values the time will be measured manually in each trial and averaged and the ratio compared to baseline measurement will be also calculated.

A multiple linear regression analysis will be performed to estimate the strength of the associations between the rMT, PPT, CPM, TS, psychological and functional variables, and pain. CPM, TS and neurophysiological variables will be used as predictors. The strength of the association will be examined using regression coefficients (B), *p* values and adjusted R2. Standardized beta coefficients will be reported for each predictor variable.

For the EEG entropy analysis non-parametric Kruskal–Wallis (KW) tests will be performed to compare the distributions, Mann–Withney U test to evaluate the difference between the median values for Pre-intervention/Controls, Post-intervention/Controls or Pre/Post intervention, and non-parametric Wilcoxon rank-sum test for Pre/Post intervention, with a significance level of 0.05 and the alternative hypothesis of unequal values. Thus, small *p*-values will suggest significant differences between populations.

The first part of the analysis will consist of comparing the differences between ON state versus OFF state in pain intensity, conditioned pain modulation, and temporal summation, and the second part will investigate the effects of the intervention on these same variables comparing the differences between ON state versus OFF state. For this analysis repetitive measures of ANOVA will be performed followed by post-hoc test with Bonferroni correction using the interaction of group factor x state factor and time factor.

### 2.6. Further Analysis

Depending on the result of each protocol a superiority trial analysis will be proposed to compare the effects of both approaches. The study will confirm the superiority of one of both protocols if the difference of the two means of King’s Pain Scale reduction is statistically significantly different at the 5% level (*p* < 0.05) two-sided and if the two-sided 95% confidence interval for the difference between the means excludes zero.

### 2.7. Dissemination Plans

All results will be published in specialized scientific journals. The final dataset from these studies will be anonymized and accessible after publication upon justified request to the main researchers. Results will be made public to the general public through the social media of our institution.

## 3. Discussion

Our studies are very relevant in the quest for an effective non-pharmacological management of pain in PD. This symptom has a high prevalence and impacts negatively in the quality of life of affected subjects. Moreover, nowadays this matter becomes even more relevant because lack of physical activity produced because of pandemic mobility restrictions could increase pain. 49.7% of Spanish PD patients report having suffered from pain every day during the COVID-19 pandemic [67].

We propose two treatment strategies whose action mechanisms are compatible with the hypothesized pain mechanisms implied in PD and have been effective in the management of pain in other chronic pain conditions. Furthermore, both approaches are proposed to be applied in combination with conventional treatments, mainly based on exercise and dopaminergic medication, in order to enhance their effectiveness through predisposing the cortex to be more easily excited. Finally, both treatments, after the configuration and explanation, can be applied at home, facilitating independence and self-management, and maximizing the time out of medical centers. Future studies may combine tDCS over M1 with AO+MI-BCI if the results of both studies are satisfactory.

Additionally, our protocols will potentially give us valuable information about pain mechanisms and enlighten new solutions for its management in PD patients. For this purpose, firstly, we propose an integral evaluation of pain, not only measuring clinical pain, but also assessing the functioning of the descending pain modulatory system, the nociceptive pathways excitability, and possible peripherical and central hyperalgesia, establishing correlations between them. Secondly, we also have the intention to figure out whether emotional, cognitive, functional, neuropsychological, or neurophysiological aspects can influence on pain and its central processing in PD patients.

## Figures and Tables

**Table 1:** Additional inclusion and exclusion criteria for the transcranial direct current stimulation (tDCS)-based Study.

Inclusion Criteria	Exclusion Criteria
Older than 18. Neuroimaging study without previous pathologies. Score > 5 in transfers (bed to chair and back) item in Barthel Index. Score = or > 24 in Mini-Mental State Examination. Tolerability for the application of electrotherapy. Able to provide informed consent to participate in the study.	History of neurologic disease different from PD. Presence of non-related to PD pain. Dermatologic problems, wounds, or ulcers in the electrode’s application area. Presence of implants or metal pieces in the head. Presence of cardiac pacemaker, vagal, brain or transcutaneous stimulators, medication pumps, ventriculoperitoneal shunts or aneurysm clips. Significative difficulties in language. History of alcohol or drugs abuse. Non-controlled medical problems. Pregnancy. Epilepsy.

PD, Parkinson’s Disease.

**Table 2:** Additional inclusion and exclusion criteria for AO+MI-based study.

Inclusion Criteria	Exclusion Criteria
Older than 18. Neuroimaging study without previous pathologies. Score > 5 in transfers (bed to chair and back) item in Barthel Index. Score = or > 24 in Mini-Mental State Examination. Able to provide informed consent to participate in the study.	History of neurologic disease different from PD. Presence of non-related to PD pain. Dermatologic problems, wounds, or ulcers in the electrode’s application area. Significative difficulties in language. History of alcohol or drugs abuse. Non-controlled medical problems. Pregnancy.

PD, Parkinson’s Disease.

## Data Availability

The data presented in this study will be available on request from the corresponding author.
